# Enhanced Dynamic Window Approach for socially compliant robot navigation

**DOI:** 10.3389/frobt.2026.1769678

**Published:** 2026-05-06

**Authors:** S. Ashwath, R. Mayank, S. Pavithra

**Affiliations:** School of Computer Science and Engineering, Vellore Institute of Technology, Chennai, Tamil Nadu, India

**Keywords:** dynamic window approach, proxemics, semantic navigation, social robotics, socially aware navigation

## Abstract

While contemporary deep learning methods are frequently computationally costly, traditional local planners like the Dynamic Window Approach (DWA) are essentially constrained by their purely geometric, “socially blind” nature. This research introduces Semantic-DWA, a unique, lightweight, and interpretable framework that closes this gap by adding a critical layer of semantic knowledge to the traditional DWA. Our methodology utilizes a perception function to categorize obstacles as “person,” “pet,” or “object” and implements a social disqualification rule that treats class-specific proxemic boundaries as hard constraints. Evaluated in a Python-based 2D simulator, comparative results demonstrated that while the standard DWA led to multiple collisions and proxemic violations, the Semantic-DWA completed all runs with zero collisions, maintaining distinct safe clearances such as 1.00 m for persons and 2.08 m for pets. This study indicates that meaningful social intelligence can be added to proven local planners through minimal extensions, offering a verifiable and predictable solution for safer human-robot coexistence.

## 1 Introduction

Autonomous mobile robots are gradually becoming part of everyday human environments, ranging from hospitals and offices to homes, and their navigation behaviour plays a crucial role in determining how comfortably people accept them. In these environments, it is not only important that robots avoid collisions; their motion should also appear intentional, predictable, and sensitive to the tacit social norms that guide human interactions. Although the Dynamic Window Approach (DWA) is considered an effective and widely used method for local planning due to its efficiency and strong geometric foundation, its traditional formulation does not differentiate between different types of obstacles encountered during navigation. Without the ability to distinguish a person from a chair or a pet, the planner may generate motions that appear abrupt or intrusive, leading to violations of personal space (proxemics) and ultimately causing discomfort or distrust among nearby humans. To address this limitation, a substantial body of research has attempted to equip robots with mechanisms that model or infer human-like navigation behaviour. For example, Inverse Reinforcement Learning (IRL) methods have been proposed to uncover the underlying decision-making patterns that humans exhibit while navigating around others, enabling robots to replicate socially compatible trajectories (Kretzschmar et al., 2020a). Other studies focus on predicting human locomotion through feature-based trajectory models, allowing a robot to estimate where interactions with humans are most likely to occur and to plan its behaviour accordingly (Kretzschmar et al., 2020b). Despite the advanced behavioural insights these approaches provide, their high computational requirements and reduced interpretability often limit their applicability in safety-critical or resource-constrained systems. Another increasingly explored approach is the incorporation of semantic information. Rather than relying solely on geometric representations, semantic navigation integrates knowledge about what the robot is interacting with in its environment. This enables the system to apply behaviourally appropriate constraints and responses. Previous work has demonstrated that semantic-assisted SLAM can utilize dynamic obstacle information to filter or reinterpret environmental features, resulting in more stable mapping (Song et al., 2025). Similar ideas have also been applied to local planning frameworks, such as Semantic Priority Navigation, where lightweight classifiers label obstacles and modify penalty functions based on their semantic category (Wang et al., 2023).

Building on these developments, the present work proposes a more interpretable extension of the classical DWA. The core idea is straightforward: provide the planner with sufficient semantic awareness to distinguish between different types of obstacles, such as people, pets, and static objects, and enforce class-specific proxemic constraints during motion planning. The framework separates perception and planning into two components. Obstacles are first assigned semantic labels by a perception module, after which the modified DWA incorporates these labels into its trajectory evaluation process. Any candidate trajectory that enters a predefined social boundary associated with a particular obstacle category is immediately rejected. In this way, social compliance is treated as a strict constraint rather than a soft optimization preference. The complete design, rationale, and implementation of the Semantic-DWA system are presented in this paper and evaluated using a lightweight Python-based simulator. The proposed approach preserves the efficiency and simplicity of the conventional DWA while introducing a modest but meaningful level of social awareness, enabling robots to navigate human environments in a safer and more natural manner.

Although semantic and context-aware robot navigation has been studied in prior work, much of the current literature focuses on learning-based decision-making models that rely on complex perception pipelines and large training datasets. These approaches typically aim to optimize navigation behaviour through end-to-end or partially learned policies. In contrast, the contribution of this work does not lie in semantic perception itself, but rather in demonstrating how minimal semantic information can be integrated into a classical local planner through a simple and interpretable mechanism. By enforcing social constraints as hard disqualification rules within the Dynamic Window Approach, the proposed method emphasizes interpretability, predictable behaviour, and compatibility with existing navigation stacks—properties that are particularly important for safety-conscious and resource-constrained robotic systems.

## 2 Related works

Research on robot navigation in human environments spans several overlapping themes, and it is helpful to look at these threads together to understand where the present work fits. Although the literature is broad, four areas have been especially influential: socially-aware navigation, improvements to the Dynamic Window Approach, learning-driven methods, and the recent movement toward semantic or context-rich planning (El Noor et al., 2025).

### 2.1 Socially-aware navigation

With robots intruding on some of the areas once filled by humans, one of the most common questions replicated in the literature is how to make them act in a way that would be considered socially sensible. This issue is also presented at length in surveys like that by Möller et al., which suggest that it is not merely sufficient not to collide with a person, but that individuals want robots to move in a manner that mimics the behavioural patterns they observe in human interactions (Möller et al., 2021). This is the same theme reflected in work on automated vehicle innovation, where researchers state that it is not only necessary not to collide with a person, but also that people expect robots to behave in a manner that reflects the patterns that they observe in every human interaction (Dong et al., 2025).

Much of such studies is dedicated to proxemics. Research on the human-robot interactions within the concrete environment, like construction sites, demonstrates that humans share relatively regular expectations regarding the proximity that has to be maintained between a robot and them (Kim et al., 2024). Other scholars have gone as far as to translate these expectations into explicit computational constraints, making social distance more of a barrier that a planner cannot cross (Jang and Ghaffari, 2024). Instead of coding social zones as continuous constraints in an optimization model (Jang and Ghaffari, 2024), the current approach uses proxemic limits as discrete feasibility filters in a reactive planner. This difference points to the role of implementing social compliance via trajectory disqualification as opposed to cost shaping. Other works consider the reactions of pedestrians to autonomous and human-driven agents differently, providing a clue of how robots ought to change their behavior to look less abrupt or unpredictable (Lanzaro et al., 2023; Boldrer et al., 2022). Combining these concepts, numerous authors believe that robots require a certain amount of natural intelligence to be effective when interacting with people, as opposed to both living and working around them (Banda and Chandra, 2025).

Different planning strategies have also been employed by navigation researchers to solve this challenge. The Velocity Obstacle (VO) scheme of reactive frameworks assists robots in observing motion conventions in relocating crowds (Thyri and Breivik, 2022), whereas optimization-based plans like MPC and dynamic-program planners aim at influencing socially acceptable paths within longer time horizons (Lin and Lin, 2024; Tran et al., 2024). One of the typical challenges in this area is that various individuals have different preferences toward the degree of distance or caution, which new research has attempted to address more explicitly (Shibata et al., 2023). Although newer methods have emerged, the DWA is still a workhorse in local motion planning as it is stable, relatively straightforward to tune, and computationally inexpensive. It has had a long life, which has promoted a continuous flow of adaptations. The field robots also receive some of these improvements, e.g., planners to navigate an orchard or an agricultural machine are based on modified versions of DWA to adapt to the uneven or semi-structured conditions (Luo et al., 2025a; Wang et al., 2025). It has been generalized by other researchers to deal with coordinated behaviours, like formations of robots (Cao and Nor, 2024), or modified to work with three-dimensional movement so that UAVs can move through narrow or congested spaces (Bes et al., 2025). The hybrid strategies also can be often found. An example is Yao et al. that use fuzzy logic and energy-related concerns as part of the classical formulation of DWA and demonstrates that the algorithm can be modified to put a higher emphasis on a variety of operational constraints without compromising its very essence of being reactive (Yao et al., 2024). This ongoing stream of update indicates that the community finds value in building up on an existing planner instead of discarding it and hence our own effort to build the same framework and add new layers of rationale to the building.

### 2.2 Recent developments to the Dynamic Window Approach (DWA)

DWA has lasted the test of time, exceeding other newer methods and ports because of its stability, relative all-controllability and light computation. Its durability has promoted the extended progressively stream of adaptations. Other of these gains are aimed at field robots, such as the planners of the orchard navigation or agricultural machinery are adapted to uneven or semi-structured worlds with modified versions of DWA (Luo et al., 2025a; Wang et al., 2025). Other authors have generalized it to deal with coordinated behaviours (e.g., formations of robots) (Cao and Nor, 2024) or modified the algorithm to support three-dimensional motion to allow UAVs to maneuver through narrow or crowded environments (Bes et al., 2025).

Hybrid methods are also common. An example is Yao et al., who leave fuzzy logic and energy to the common DWA formulation and demonstrate that by modifying the method, it is possible to embody various restrictions of operation to still retain its primary reactive nature (Yao et al., 2024). This endless stream of updates indicates that the community believes in adding to an already existing planner instead of abandoning it and that is why such endeavors as our one are driven by the principle not to break the backbone of the structure and only add some additional layers of argumentation.

### 2.3 Learning-based navigation

Other significant bodies of work involves learning-based approaches to tackling the navigation problem in changing environments. Deep Reinforcement Learning has gained popularity especially in those cases when the behaviour needs to adapt to rapidly changing environments. DRL has been used to solve the problem of training autonomous vehicle navigation policies (Sivayazi and Mannayee, 2024) and to optimize reward signals to get smoother or more robust trajectories (He et al., 2025). DRA versions have also been applied to marine robotics, where the agent has to deal with uncertainty in complicated water conditions (Huang et al., 2025). An analogous method, Inverse Reinforcement Learning, attempts to learn human preferences by observation of behavior. As an illustration, IRL has been applied to explain the behavior of road users with regards to autonomous cars, which aids the robots to imitate socially acceptable reactions (Alozi and Hussein, 2024). Although these learning-based systems are admittedly powerful, their decision-making is usually opaque internally-a problem when the robot has to defend or clarify their actions in human settings.

**FIGURE 1:**
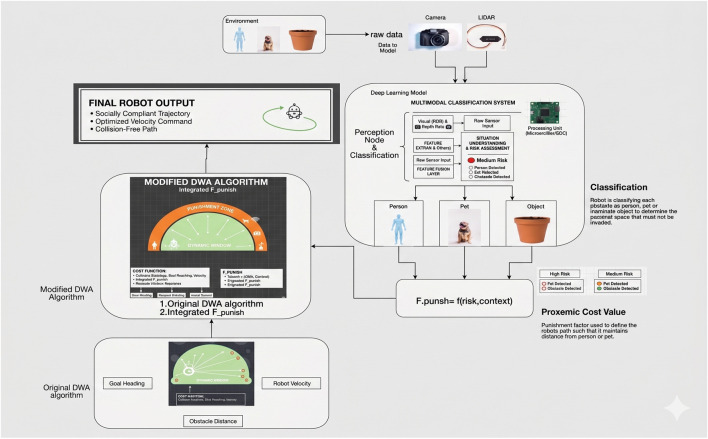
Proposed System architecture.

The conceptual representation of the sequential flow of data among these components, between the perception of the environmental and the final velocity command, is presented in Figure 1. The essence of this integrated perception and planning cycle is proposed in a consolidated form in Algorithm 1, which has every step further explained in the following subsections.

### 2.4 Semantics and context in planning

The more current trend of the field has been to move toward utilizing semantic information as opposed to merely utilizing geometric maps. A good example is semantic segmentation, which has allowed robots to explore areas such as orchards without GPS based on meaningful labels found on camera information (Navone et al., 2025). The same has been applied to tasks. As when creating the navigation line automatically in an agricultural scene (Luo et al., 2025b) or improving SLAM pipelines so that moving objects do not corrupt the map (Chen et al., 2025).

An increasing role in higher-level tasks is also taken by semantic cues- Inspection or monitoring robots, such as those performing critical tasks, also use the categories of objects to interpret the perceived image and make a choice (Hu and Gan, 2025). The recent article goes so far as to discuss how to wire big language models into the navigation systems could make robots be able to read into the social circumstances and contextual indicators more profoundly (Lamichhane et al., 2025). All this leads to a larger tendency: planners incorporating some information about what lies in the environment tend to act smarter than those which assume everything is a nameless difficulty. The current research proceeds in this concept in a lighter and easier way by integrating semantic labels and the already established DWA. A number of the current social navigation methods incorporate social awareness in terms of soft cost functions or force-based interaction models, like the Social Force Model or layered cost map techniques which are frequently employed in ROS-based social navigation systems. These approaches do not outlaw socially undesirable behaviour, but rather penalise it. We deliberately limit the sphere of the experiment in this work to the standard Dynamic Window Approach so that we only isolate the effect of the application of semantic constraints into the same planning framework. A comparison with fundamentally different planners or learning based systems would bring more assumptions of perception pipelines, training data and parameterization and it may not be simple to attribute behavioural change to the proposed semantic disqualification mechanism (Helbing and Molnár, 1995).

## 3 Proposed methods

This section comprehensively details the proposed framework for socially-aware robot navigation, which integrates a perception function for semantic classification, a social disqualification rule for proxemic awareness, and a modified Dynamic Window Approach (DWA) local planner implemented in a lightweight Python simulation.

### 3.1 Algorithm 1: semantic-DWA simulation cycle

Algorithm 1 Semantic-DWA Simulation Cycle.

Input:
 Current_Robot_State
 Goal_Position
 List_of_All_Obstacles
Output:
 Optimal_Velocity_Command
Possible_Trajectories ← GENERATE_TRAJECTORIES(Current_Robot_State)
Min_Cost ← ∞

Best_Velocity ← Current_Robot_State.velocity
for each trajectory in Possible_Trajectories do
 is_socially_invalid ← FALSE
 for each obstacle in List_of_All_Obstacles do
  min_dist ← CALCULATE_MIN_DISTANCE(trajectory, obstacle)
  if obstacle. type = = “pet” and min_dist < PET_THRESHOLD then
   is_socially_invalid ← TRUE
   break
  else if obstacle. type = = “person” and min_dist < PERSON_THRESHOLD then
   is_socially_invalid ← TRUE
   break
  else if (obstacle.type = = “object” or obstacle. type = = “distractor”)
    and min_dist < OBJECT_THRESHOLD then
   is_socially_invalid ← TRUE
   break
  end if
 end for
 if is_socially_invalid = = TRUE then
  continue
 end if
 goal_cost ← CALCULATE_GOAL_COST(trajectory, Goal_Position)
 obstacle_cost ← CALCULATE_OBSTACLE_COST(trajectory, List_of_All_Obstacles)
 speed_cost ← CALCULATE_SPEED_COST(trajectory)
 total_cost ← goal_cost + obstacle_cost + speed_cost
 if total_cost < Min_Cost then
  Min_Cost ← total_cost
  Best_Velocity ← GET_VELOCITY_FROM_TRAJECTORY(trajectory)
 end if
end for
if Best_Velocity is unchanged then
 Optimal_Velocity_Command ← ZERO_VELOCITY
else
 Optimal_Velocity_Command ← Best_Velocity
end if
return Optimal_Velocity_Command

### 3.2 System architecture and data flow

**FIGURE 2:**
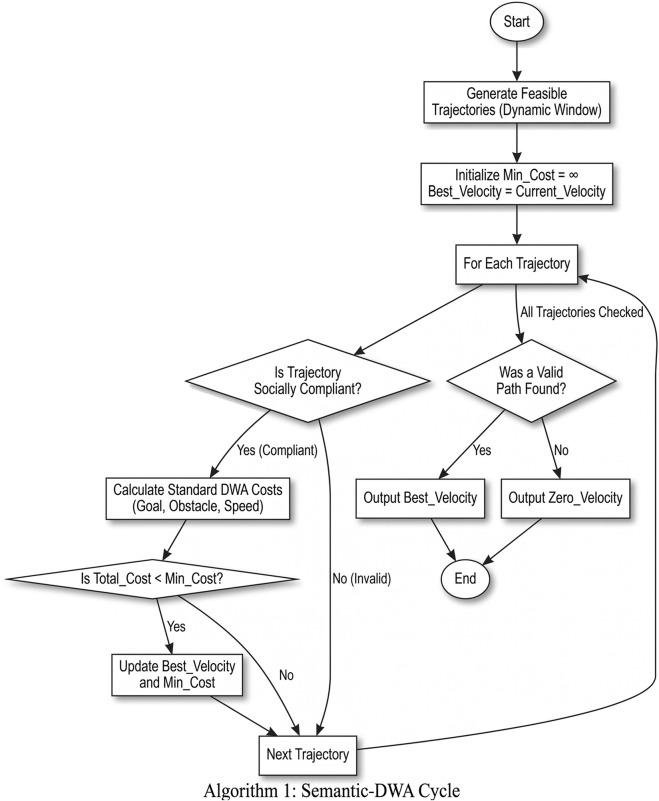
Dynamic window approach process flow.

Figure 1 sketches the overall layout of the system, which is implemented in a lightweight Python simulator which is expressed in 2D. The environment of the simulation has a simple world model consisting of a single robot and a list of obstacles which move or stay still according to their assigned behavior. The obstacles are instantiated with three major properties, namely, position, the movement pattern (only in the case of a moving obstacle), and a semantic tag which can be a person, pet, or object. These labels are ground-truth classifications to the planner. The simulator does not construct a complete perception pipeline, as would be required under the scope of this study, but the planner has direct access to this list of obstacle objects. This arrangement allows us to isolate and test the navigation logic in isolation without sensor modelling noise and uncertainty. At each simulation cycle, the modified DWA planner takes the list as an input. The semantic information is used by the planner to filter the trajectories and then the standard geometric scoring is applied, so that the socially unacceptable motions do not reach the optimization part. After the planner has settled on the optimal candidate trajectory, the respective velocity command is fed back to the simulation loop. This command is then used to update the position of the robot and the procedure continues. The outcome is a clean and transparent pipeline with perception (simplified) as input having a direct connection to a socially conscious DWA planner which then commands the movement of the robot. The same has been illustrated in Figure 2.

### 3.3 Perception and social obstacle classification

Since the semantic type of all obstacles is already present in our simulation environment, classification is no longer a separate module, but a part of the planning loop. Each time the planner starts a cycle, he or she analyzes each viable path and ensures that each path collides with all the obstacles within the environment. Each obstacle will be found in the algorithm, the. type label is returned, and the minimal distance between the obstacle and the candidate trajectory is calculated. The distances are then contrasted with class related thresholds-a personal space boundary of a person is not the same as the one of a small pet or an immobile object. When a trajectory is found to cross any of these specified limits, then it is indicated as socially invalid and it is eliminated. The set up forms some kind of ideal perception, which is intended. This work addresses the issue of the behavior of a planner when semantic understanding is known, rather than the process by which robots learn the labels. By including the classification process directly into the trajectory-screening process it is guaranteed that the cost functions never consider any path that does not meet the physical safety criterion as well as the social comfort criterion.

#### 3.3.1 Proxemic threshold selection

The Semantic-DWA planner enforces class-specific minimum distance thresholds that reflect commonly reported proxemic norms in human–robot interaction literature. In the experiments presented in this study, the following thresholds were used:

- Person: 1.0 m, corresponding to the personal–social space boundary reported in proxemics theory and observed in empirical HRI studies.
•Pet: 2.0 m, reflecting higher uncertainty and reduced predictability in motion compared to adult humans.
•Static Object/Distractor: 0.6 m, consistent with conservative clearance margins commonly used in mobile robot navigation.

These values were selected to be representative rather than optimal, and the framework itself remains agnostic to the specific thresholds, allowing application-dependent tuning without modifying the planner logic (Hall, 1966).

### 3.4 Socially-aware Dynamic Window Approach (Semantic-DWA)

The altered DWA planner has an identical structure as the classical algorithm but includes an important filtering procedure. Once the planner has generated a number of viable trajectories within the dynamic window, each of them is sent through the social disqualification check as outlined above. This implies that the proxemic constraints are turned into constraints that are hard-to-meet, any path that crosses into a social zone is just discarded. After such filtering only does the planner calculate the typical DWA costs, including goal-directedness, clearance of obstacles as well as forward speed. The planner also does not deal with the situation in which the most geometrically optimal solution could be socially undesirable; this is because the planner has eliminated the socially compliant trajectories, first. The result of this filtering is just the cheapest path through this set or, when a valid path is not identified, a zero-velocity program which leads the robot to halt. This makes the robot have an intuitive behavioural characteristic, that it moves easily when the path is clear but slows or turns around itself when a person or pet is present in the pathway, despite the existence of a technically collision free shortcut path.

### 3.5 Mathematical formulation

This section formalizes the cost functions that guide the planner’s decision-making process. We first present the objective function of the classic DWA before detailing our novel social disqualification rule and its integration into the modified planner.

#### 3.5.1 Traditional DWA cost function

The traditional Dynamic Window Approach (DWA) selects the optimal velocity command (v,ω) by scoring a set of feasible trajectories. The score for a given trajectory, G(v,ω), is a normalized, weighted sum of several competing objectives. The function is typically expressed in Equation 1:

$$G\left(v,\omega \right)=\sigma \left(\alpha \cdot \text{heading}\left(v,\omega \right)+\beta \cdot \text{dist}\left(v,\omega \right)+\gamma \cdot \text{vel}\left(v,\omega \right)\right)$$ (1)

where:

v and ω are the linear and angular velocities.

heading(v,ω) is the cost associated with the robot’s alignment to the goal direction.

dist(v,ω) is the cost representing the distance to the nearest obstacle.

vel(v,ω) is a utility function that rewards maintaining a high forward velocity.

α,β, and γ are weighting parameters that balance these objectives.

σ is a normalization function to scale the total score.

As shown in Equation 1, this formulation is purely geometric and does not account for the semantic type of the obstacles it avoids.

#### 3.5.2 Semantic-DWA social disqualification rule

Our primary contribution is the replacement of a simple additive penalty with a Social Disqualification Rule, applying constraint based on the semantic class of an obstacle. Rather than computing an extra penalty, we check whether a trajectory is a socially compliant one or not. The trajectory, traj, is deemed compliant when at all the points p on the trajectory the distance to all the categorized social obstacles o in the set Osocial is furthermore larger than or equals the proxemic threshold D of a given type of obstacle. This condition is expressed in Equation 2:

$$\text{is}\_\text{socially}\_\text{compliant}\left(\text{traj}\right)=\forall o\in \text{Osocial},\forall p\in \text{traj}:d\left(p,o\right)\geq \text{Do}.\text{type}$$ (2)

where Do. type is the predefined proxemic threshold (PERSON_THRESHOLD, PET_THRESHOLD, etc.) for the semantic class of obstacle o.

The final cost of the trajectory is then determined by a piecewise function that either applies the standard DWA cost or disqualifies the path entirely. This is shown in Equation 3:

$$\text{Cost}\_\text{modified}\left(\text{traj}\right)=\left\{\;G\right.\left(\text{traj}\right),\text{if traj is socially compliant}$$

$$\left\{\;|\infty ,\text{otherwise}\right.$$ (3)

**FIGURE 3:**
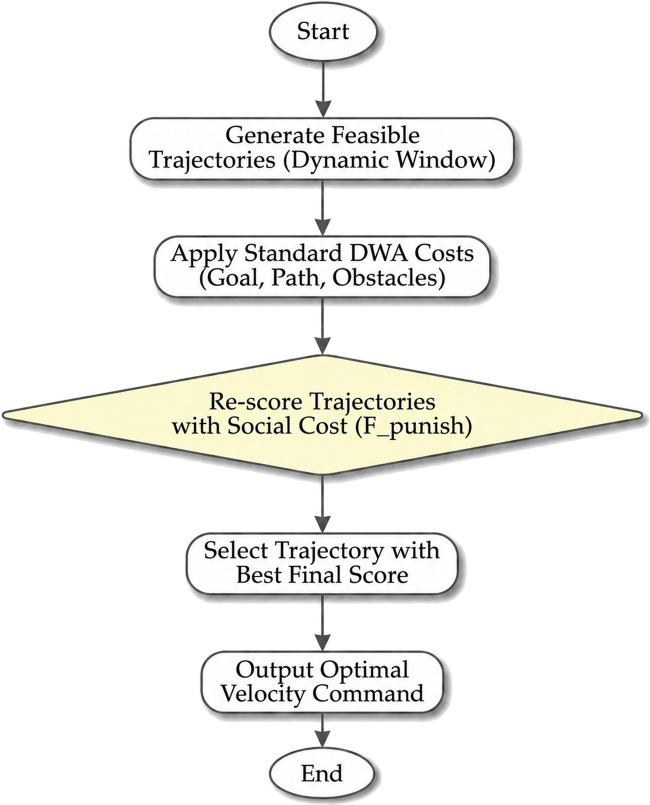
Semantic Enhanced-DWA flowchart.

This is a stipulation of the social threshold such that any route with a cost exceeding a social threshold is automatically disqualified as shown in Equation 3. The job of the planner is to then identify the least cost path out of the now down-selected set of socially compliant options. This causes the social rules that were soft suggestions, to be hard constraints, to ensure that the behavior is compliant whenever there is a valid way of doing things. The entire process can be seen in Figure 3.

## 4 Experimental set up and evaluation metrics

To confirm the effectiveness and functionality of our intended Semantic-DWA framework, we performed a set of comparative experiments in a simple, repeatable 2D simulation, which could be replicated. The overall goal was to evaluate our modified planner quantitatively and qualitatively with reference to the standard DWA.

### 4.1 Experimental setup

For measuring the Semantic-DWA behavior in comparison with the standard version of the planner, we constructed a simple yet controlled simulation space in Python (NumPy and Matplotlib) and ran all experiments on Google Colab. The intention of the arrangement was not a high-quality physics simulation of high fidelity but to offer a clean and reproducible environment in which the differences in behaviour could be easily observed. The simulated world is a 20 × 20 m square. The robot begins at coordinates (2.0, 2.0) and is instructed to reach a target placed diagonally across the map at (18.0, 18.0). Along this path, we placed four obstacles designed to reflect common interaction types in everyday environments:

- A “person” that moves back and forth along a vertical line.
- A “pet”, which follows a more erratic, curved pattern to mimic the unpredictable movement of a small animal.
- A stationary object, positioned directly in what would otherwise be the robot’s shortest route to the goal.
- A horizontal “distractor”, which moves side to side but does not represent a social entity.
- Each obstacle’s motion and label remain consistent throughout the experiment, allowing both planners to be tested under identical conditions.

Two planner configurations were compared:

- Standard DWA, which uses only geometric criteria such as distance from obstacles, heading toward the goal, and forward speed.
- Semantic-DWA, which includes the social disqualification rule and applies class-specific proxemic boundaries before the usual cost evaluation.

This setup ensures that any differences in performance can be traced back to the presence or absence of semantic filtering. For fairness, both planners were configured with identical Dynamic Window parameters. The linear velocity range was set to [0, 1.0] m/s and angular velocity range to [−1.0, 1.0] rad/s. The trajectory simulation time horizon was 2.0 s with a resolution of 0.1 s. Cost weights were set to α = 0.6 (goal heading), β = 0.2 (obstacle clearance), and γ = 0.2 (velocity preference). These parameters were tuned conservatively to avoid biasing either planner toward aggressive motion.

### 4.2 Assumptions and scope of evaluation

In this research, the semantic labels of the obstacles are supposed to have the simulation environment as providing the ground truth. Such a design decision was selected with the purpose of isolating and testing the behaviour of the proposed Semantic-DWA planner, without considering the uncertainty in perception. In practical navigation, perception errors including misclassification, missed detections and noises at the sensor are an important cause of failure. Nevertheless, these aspects would ultimately mix the judgment logic of the planner with perception-related biases. The work does not take into account the implementation of the planning layer and the influence of imposing semantic social constraints as hard disqualification rules due to the assumption of perfect perception.

The suggested approach has great potential as future directions include extending its functionality to deal with noisy perception pipelines, e.g., those using vision or LiDAR-based semantic segmentation, and testing the method on real robots, e.g., in physics-based simulators (e.g., ROS/Gazebo). In a real deployment, it is possible that the misclassification or wrongful omission can cause incorrect thresholding to be assigned, which in turn can cause overly conservative behaviour or inadequate clearance. The suggested framework is however, modular: the semantic labels are absorbed as external input, and the uncertainty-aware perception models or probabilistic classification confidence scores might be added without changing the structure of the planner. As an illustration, when the confidence in classification is weak, one may employ conservative fallback thresholds and ensure safety in times of uncertainty.

### 4.3 Evaluation metrics

We assessed the planners using a combination of quantitative and qualitative measures:

- Collision Check (Safety).

Throughout the simulation, the robot is modelled with a physical radius of 0.5 m. A run is marked as a collision if the robot’s boundary intersects the position of any obstacle at any point. This binary measure provides a clear indication of basic safety.

- Minimum Clearance Distance (Social Compliance).

For each obstacle, we recorded the smallest distance the robot came within during its entire path. These values show how well the planner respects personal and social space, especially for dynamic entities like the person or pet.

- Trajectory Plots and Animations (Qualitative).

The robot’s motion visually often reveals patterns that metrics alone cannot capture. We generated animations of both planners’ trajectories, allowing a side-by-side comparison of behaviour, particularly useful when evaluating whether a motion appears “socially acceptable.”

Overall these metrics gave a balanced view of the robot’s performance, capturing whether it reached the goal and also how it moved through the shared space.

## 5 Results and discussion

To validate our proposed Semantic-DWA framework, we conducted a comparative simulation study against the baseline Standard DWA. This section presents and analyzes the quantitative and qualitative results from these experiments, demonstrating the efficacy of our approach in producing socially compliant and safe navigation.

### 5.1 Baseline performance: standard DWA

**FIGURE 4:**
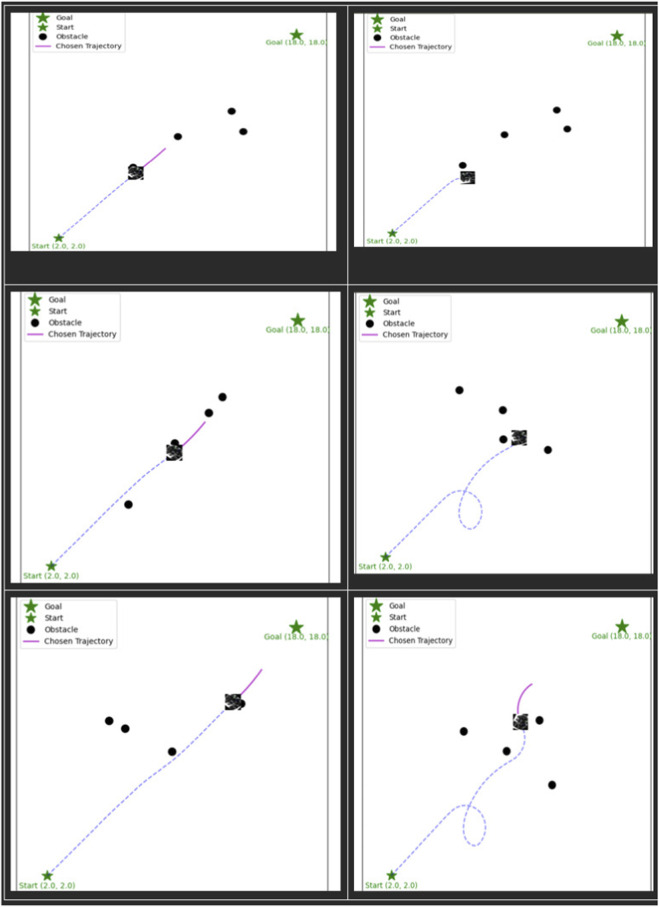
Comparison of Standard DWA(Left) and our Semantic (context-aware) DWA(Right) in collision avoidance.

The limits of the baseline DWA were revealed several seconds after we ran it through the test environment. Since the planner simply considers geometric aspects, it also gives preference to paths that aggressively approach the goal, in cases where a slower and more socially responsible route is possible. During our tests, this behaviour led to three different collisions, the first one with the individual, the second one with the pet, and the third one with the object present in the line of movement. This tendency is supported by the minimum clearance distances obtained. The robot went through a distance of ≥0.35 m of the person and >0.36 m of the pet, both of which were well within the physical radius of the robot (0.5 m). It is not surprising that the straight-line plot in Figure 4: Standard DWA—Left panel depicts a path that cuts straight through the environment without paying attention to the fact whether the obstacles are dynamic or socially sensitive. This complements the design of the planner: it does not make any distinction between the worth or the susceptibility of all the obstacles, it lacks a system to explain how they are important or not.

### 5.2 Proposed method performance: semantic-DWA

In contrast, the Semantic-DWA demonstrated a markedly different style of movement. With the social disqualification rule activated, the planner immediately ruled out any trajectory that encroached on the proxemic boundaries of the person, pet, or other obstacles. As a result, the robot completed its run without a single collision.

The clearance distances reflect the planner’s more nuanced behaviour:

- 1.00 m minimum distance from the person
- 2.08 m minimum distance from the pet
- 0.87 m from the static object
- 0.76 m from the distractor

Not only is this a safe distance that is well beyond the physical limit, but these figures demonstrate that the individual who was planning has distinguished between the types of obstacles. As an example, it maintained the biggest margin surrounding the pet - in line with the hypothesis that pets may move haphazardly - with much closer clearance in the steady object. This behaviour is depicted by the trajectory plot where the robot traverses more circles around a dynamic and socially relevant object and fewer circles around stationary objects. In the assessment, the zero-velocity fallback case occurred in case there was no socially acceptable trajectory in the dynamic window. In the experimental case, it was observed temporarily in the situations when there was close dynamic interaction but this never led to sustaining deadlock. Within more congested settings, relaxation strategies, including the gradual decrease of the threshold or the time-based recovery behaviour may be added to prevent the long cessation.

### 5.3 Comparative analysis

The quantitative data, summarized in Table 1, provides a clear and compelling comparison between the two approaches.

**TABLE 1:** Summary of performance metrics.

Metric/obstacle	Standard DWA	Semantic-DWA (proposed)
Collisions detected	YES (3)	NO (0)
Min clearance: person	0.35 m	1.00 m
Min clearance: pet	0.36 m	2.08 m
Min clearance: object	0.47 m	0.87 m
Min clearance: distractor	1.74 m	0.76 m

The summary of the results is provided in Table 1, but one can speak about the bigger conclusions. To start with, the given approach does not only play on the safe side. The fact that the clearance among types of obstacles is not universal indicates that the robot is not an omnivorously cautious robot, but a selectively protective robot. That is exactly what one would desire in a robot that would possess some batteries on what is happening around it. Second, the Semantic-DWA is not permissive of any collisions, but by laxing absurdly, or overreacting, instead of preventing socially problematic trajectories in the first place. This transformation has a significant impact on the decision-making environment of the planner: the planner will no longer be required to trade-off goal progress and solving obstacles; the system cannot even consider the paths that have already fulfilled the minimal social conditions. Lastly, the behavioural enhancement proves that relative light modifications can be made to achieve meaningful social compliance. The algorithm is not based on robust prediction models or deep learning, just using semantic labels and proxemic constraints at the stage of trajectory-filtering. Through the simplicity of this method, it makes a good case in support of semantic context as a natural follow-up of classical planning.

**FIGURE 5:**
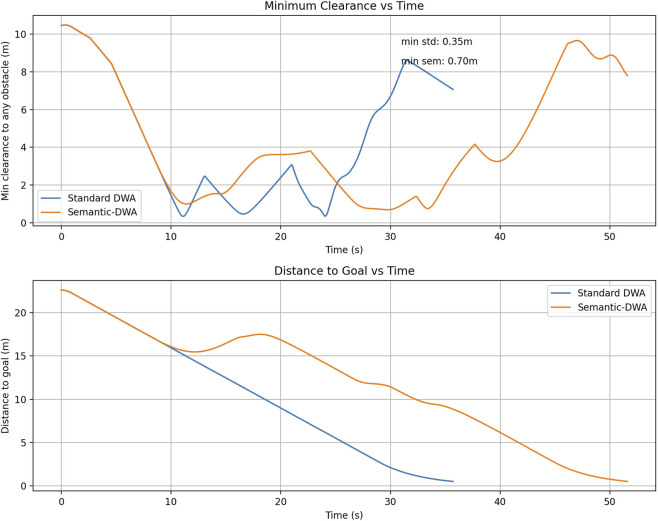
Comparison Graph between standard and Semantic DWA.

It is necessary to add that the test was performed in a controlled environment where the trajectories of obstacles were constant. Although this facilitates direct comparison of behavior, statistical validation of behavior in larger obstacle configurations that are randomized would further bolster generalizability. This is long-term assessment that will be done in future. The visualisation for this analysis can be seen in Figure 5.

## 6 Conclusion

This is an attempt to fill an ancient lapse in classical local planners: they do not know what they are heading around. Standard DWA is a fast and reliable treatment, but all obstacles are treated the same way, and our baseline experiments mirror this weakness. The planner chose socially inappropriate geometrically efficient trajectories in the presence of people, pets, and stationary objects placed at different position and moving at different velocities, resulting in collisions between them. The alternative presented here is the Semantic-DWA framework which is a simple but effective framework. Including semantic labels and using hard constraints of proxemic boundaries on the classes, the planner receives one more reasoning layer without the trade-off of the transparency and computational efficiency of the original procedure. This change can be perfectly simulated, as the robot successfully cleared its navigation without any collisions and displayed behaviour that was more in line with human expectations of personal space and comfort. Significantly, the method can be interpreted and checked. In contrast to the learning-based systems that have opaque decision-making processes, Semantic-DWA explicitly eliminates paths that cross social boundaries prior to considering the remaining candidates using cost terms in the traditional way. This renders it a viable measure towards an environmentally conscious navigation in an environment that demands predictability and explainability. Generally, the findings point to the fact that classical planners can be enhanced into being more socially intelligent by making small extensions. Although tested in a controlled simulation with perfect semantic perception, this simplification separates the behavior of planners; future analysis will include the addition of noisy perception and test the methodology in a realistic and real-world setting.

## Data Availability

The Data files for all code used in this research and related fields are available on this GitHub repository at: https://github.com/Ashwath-Shivram-22BAI1188/22BAI1188_ASHWATH_SHIVRAM_22BAI1118_MAYANK_RAJ.
